# Compound glycyrrhizin combined with antihistamines for chronic urticaria: A protocol for systematic review and meta analysis

**DOI:** 10.1097/MD.0000000000021624

**Published:** 2020-08-14

**Authors:** Wei Cao, Xianjun Xiao, Leixiao Zhang, Ying Liu, Lu Wang, Zihao Zou, Yue Cao, Chunxiao Li, Qianhua Zheng, Siyuan Zhou, Ying Li

**Affiliations:** a Acupuncture and Tuina School, Chengdu University of Traditional Chinese Medicine; b Rehabilitation Department, The People's Hospital of Jianyang City; c Dermatological Department, Affiliated Hospital of Chengdu University of Traditional Chinese Medicine, Chengdu, Sichuan, China.

**Keywords:** chronic urticaria, compound glycyrrhizin, CG, antihistamine, protocol, systematic review

## Abstract

**Background:**

To investigate the efficacy and safety of compound glycyrrhizin (CG) combined with antihistamines in the treatment of chronic urticaria (CU).

**Methods:**

We will use computers to search all databases including Medline, Embase, Pubmed, Web of Science and Cochrane Central Register of Controlled Trials and China's 4 databases: China National Knowledge Infrastructure Database, China Biomedical Literature Database, China Science Journal Database, and Wanfang Database. Find data from creation date to July 2020. In addition, we will manually search the list of medical journals as a supplement. The scope of the search included randomized controlled clinical studies related to CG combined with antihistamines for CU. The primary outcome is the disease activity control. Secondary outcomes include response rate, adverse events, and recurrence rates. The Cochrane RevMan V5.3 Deviation Assessment Tool will be used to assess bias assessment risk, data integration risk, meta-analysis risk, and subgroup analysis risk (if conditions are met). The average difference, standard mean difference, and binary data will be used to represent continuous results.

**Results:**

This study will comprehensively review the existing evidence on CG combined with antihistamines for CU.

**Conclusion:**

This systematic review will provide a basis for judging the effectiveness and safety of CG combined with antihistamines in the treatment of CU.

**Systematic review registration:**

PROSPERO, CRD42020156153

## 1 Introduction

### 1.1 Description of the condition

Chronic urticaria (CU), defined as the recurrent occurrence of wheals with or without angioedema for longer than 6 weeks, Attack twice a week or more,^[1]^ has a global incidence rate of about 1%,^[2,3]^ mainly affecting young and middle-aged women.^[4–6]^ CU can be classified into chronic spontaneous urticaria and chronic inducible urticarias which based on the absence or presence of identifiable physical stimuli able to elicit the skin lesions.^[7]^ Among them, the incidence of chronic spontaneous urticaria is about twice that of chronic inducible urticarias.^[8]^ The pathogenesis of CU may be related to multiple mechanisms, including autoimmunity, allergy and blood coagulation, each mechanism may have a different weight in each patient.^[9]^ The quality of life of patients with CU is considerable. People with CU often suffer from fatigue, pain, lack of sleep or insomnia due to persistent itching, according to research data.^[4,10]^ Visible damage can lead to emotional distress and withdrawal from social activities,^[11]^ as well as psychological complaints such as anxiety, depression, or irritability.^[10,12,13]^ It has a great impact on the life and work of patients with CU.

According to the consensus ^[14,15]^ and the guidelines ^[1,16,17]^ published these years, the initial treatment for CU is second-generation H1 antihistamines (sgAH).^[1]^ If there is no improvement after 2 to 4 weeks, the dose can be increased to 4 times the manufacturer's recommended dose. Omalizumab should be added when long-term use of sgAH fails to achieve the desired results. If there is still no improvement in half a year, it is recommended to use cyclosporine and sgAH treatment.^[17–19]^ In addition, there may be a high financial burden through direct medical costs and loss of productivity due to symptoms.^[20]^

### 1.2 Description of the intervention

Glycyrrhiza uralensis (Gan cao) is one of the key herbs used in Chinese medicine, its main active ingredient is glycyrrhizin.^[21]^ At the same time, Glycyrrhizin as the main component, together with aminoacetic and methionine, constitute compound glycyrrhizin (CG). CG has been used to treat abnormal liver function in chronic hepatitis for more than 30 years in Japan, it is also known as Stronger Neo-Minophagen C^[22,23]^ Studies have shown that glycyrrhizin not only has significant anti-inflammatory, anti-allergy, and anti-ulcer activities, but also has immunomodulatory and antiviral effects.^[24]^ It is metabolized into glycyrrhetinic acid in the body. It is similar to glucocorticoid in structure, but has few adverse reactions to steroids.^[24]^ Therefore, in recent years, glycyrrhizin has been widely used in clinical treatment of skin diseases, such as urticaria, dermatitis, alopecia areata, psoriasis, vitiligo, and so on.^[25–28]^

Antihistamines, specifically H1 antihistamines, are the main treatment for CU. The root cause of CU cannot be determined,^[29,30]^ but many CU patients have circulating antibody that is able to bind to mast cells, thereby causing histamine release and weal formation.^[31]^ And Antihistamines control symptoms just by competing for receptors of corresponding target cells, so they control the condition rather than cure it.^[31]^ Therefore, most patients need long-term medication. Studies have shown that long-term use of antihistamines can cause many adverse reactions such as headaches, drowsiness, fatigue, dry mouth and so on ^[32–34]^

Therefore, in recent years, CG combined with antihistamines has been widely used in clinical treatment of CU. Two systematic reviews (SRs)^[35,36]^ have shown that, compared with a single medication, CG combined with cetirizine can significantly improve the symptoms and signs of CU with fewer adverse reactions. In summary, a more comprehensive SR of CG combined with antihistamines for CU is needed.

## 2 Methods and analysis

### 2.1 Study registration

This research registration strictly follows the systematic review and meta-analysis plan (preferred reporting items for systematic reviews and meta-analysis protocols) preferred report item.^[37]^ And has been registered in Prospero (ID: CRD42020156153).

### 2.2 Ethics and dissemination

The results of this SR are to evaluate the published randomized controlled trials (RCT) on the efficacy and safety of CG combined with antihistamines in the treatment of CU, so as to help clinicians and patients choose appropriate treatment options. This review does not require ethical approval and will be published in a peer-reviewed journal.

### 2.3 Search strategy

We will use computers to search all databases including Medline, Embase, Pubmed, Web of Science and Cochrane Central Register of Controlled Trials and China's 4 databases: China National Knowledge Infrastructure Database, China Biomedical Literature Database, China Science Journal Database, and Wanfang Database. Find data from creation date to July 2020 by using the following search terms: Urticaria, Chronic urticaria, Nettle-rash, Hives, Rubella, Wind cluster, Angioedema, Compound Glycyrrhizin, Glycyrrhizin, Gan cao, Glycyrrhizic, Glycyrrhiza uralensis, Antihistamine, Antihistaminic, H1-antihistamine. Boolean operators “and” and “or” will be used within the search to combine the search terms. The example search strategy in Table 1 will be used for Pubmed. The search strategy for each of the other sites is adapted to the characteristics of the database. We will search the list of related references for more trials and existing SRs related to our topics by PubMed and Cochrane Library, and will also search a reference list to identify published journals related to the research topic, Books, conference articles, and grey literature.

**Table 1:**
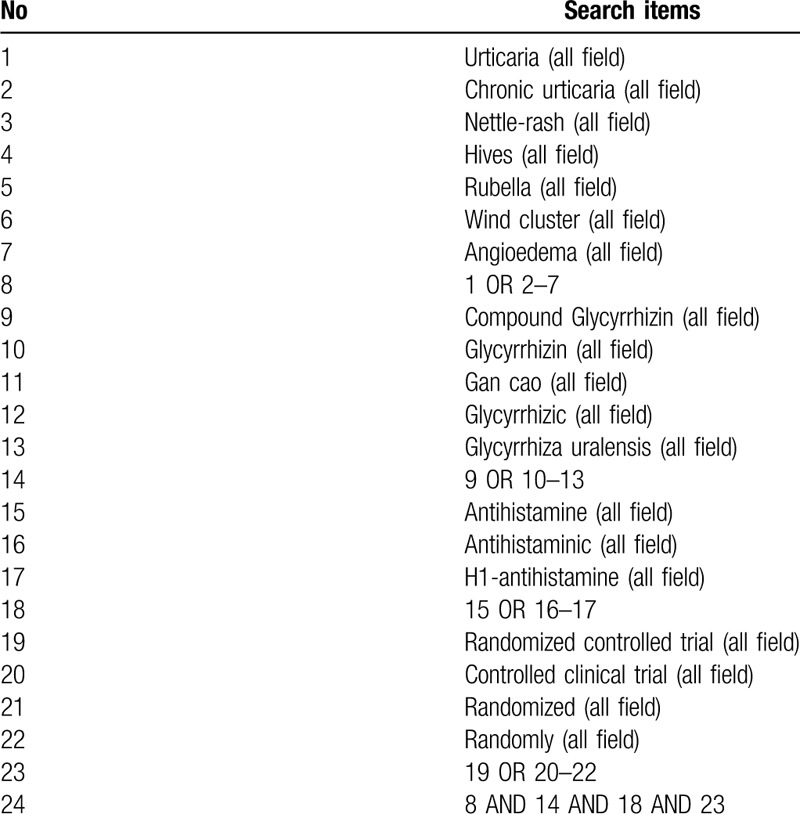
The search strategy used in PubMed.

### 2.4 Criteria for including studies

#### 2.4.1 Types of studies

This article only reviews the RCT of CG combined with antihistamines as the main treatment. The control group included no treatment, placebo, separate antihistamines, and other effective treatments. In addition, both Chinese and English publications are subject to language restrictions. RCTs that are not subject to release status will be included, excluding the remaining types of documentation.

#### 2.4.2 Types of participants

No matter what race, gender, age, and education, in our SR patients must comply with the European Academy of Allergology and Clinical Immunology, the Global Allergy and Asthma European Network, World Allergy Organization (EAACI/GA2LEN/EDF/WAO) guidelines ^[1]^ or the Chinese guidelines for the diagnosis and treatment of urticaria version.^[38]^

#### 2.4.3 Types of interventions and comparisons

Qualified interventions in the experimental group can only be combined with CG and antihistamines, which will be excluded if combined with other methods (such as traditional Chinese medicine decoction, acupuncture, etc) or a separate drug. The following processes will be compared:

(1) CG combined with antihistamines therapy compared with no treatment.
(2) CG combined with antihistamines therapy compared with placebo or sham drugs therapy.
(3) CG combined with antihistamines therapy compared with a separate antihistamines therapy.
(4) CG combined with antihistamines therapy compared with other active therapies.

#### 2.4.4 Types of outcomes

##### 2.4.4.1 The primary outcomes

The primary outcome was disease activity control, measured by the urticaria activity score, urticaria control test, or other validated symptom scores.

##### 2.4.4.2 The secondary outcomes

(1) Response rate.
(2) Recurrence rate during the follow-up period.
(3) Adverse events.

### 2.5 Data collection and analysis

#### 2.5.1 Selection of studies

**Figure 1:**
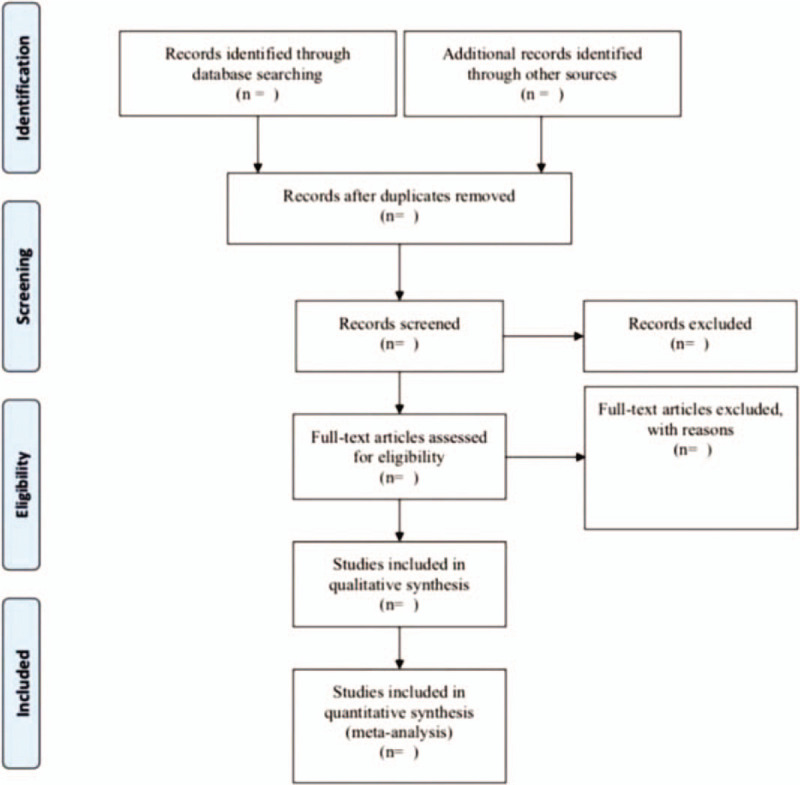
The PRISMA flow diagram of the study selection process. PRISMA = preferred reporting items for systematic reviews and meta-analysis protocols.

The references in the search results will be added to the EndNote software (V.X8) document management software, and the duplicate content will be deleted. The 2 review authors (XX and LZ) will conduct an independent screening of the titles, abstracts, and keywords of all search studies to confirm qualified trials. Reviewers will receive a complete report for further evaluation. The excluded explanations will be recorded in the excel dataset. If the differences between the reviewers (XX and LZ) are not resolved through discussion, third-party arbitration (YC) determines. The research flow chart is shown in Figure 1.^[39]^

#### 2.5.2 Data extraction and management

The 2 authors (LW and QZ) will extract data independently from the selected report or study and fill out the data extraction form. Extract the following information: general information, participants, methods, interventions, results, adverse events, main conclusions, conflicts of interest, ethical approval, and so on. When the reported data is insufficient, we will contact the author for more information. In this process, any inconsistencies will be resolved through discussion between the 2 authors or judged by the third author (YL).

### 2.6 Assessment of risk of bias in included studies

The 2 authors (WC and QZ) will evaluate the quality of the research by using a list of risk assessment tools by Cochrane Collaborative bias, to evaluates the presence of potential selection bias (random sequence generation and allocation concealment), performance bias (blinding of investigators and participants), detection bias (blinding of outcome assessors), attrition bias (incomplete outcome data), reporting bias (selective reporting), and possible other sources of bias (funding bias). This review uses L, U, and H as the key to these assessments, where L (low) indicates a lower risk of bias, U (unclear) indicates that the risk of bias is uncertain, and H (high) indicates a higher risk of bias. If inconsistent results appear, the third author (SZ) makes the final decision.

### 2.7 Measures of treatment effect

For dichotomous data, risk ratio with 95% confidence intervals (CIs) will be used to measure the treatment effect. For continuous data, mean difference and 95% CIs were used to measure treatment effectiveness.

### 2.8 Dealing with missing data

We will handle with the missing data according to the Cochrane Handbook guidelines for systematic review interventions. In particular, the following methods will be used:

(1) Contact the corresponding author for missing data requests.
(2) Execution analysis is available.
(3) Discuss the potential impact of lost data.

### 2.9 Assessment of heterogeneity

We will evaluate the statistics of heterogeneity through Cochran's Q test and quantify them through I2 value.^[40]^ If the results are statistically significant, the reasons will be discussed through narrative, subgroup analysis, and sensitivity analysis.

### 2.10 Assessment of reporting biases

We will evaluate publication bias using the Egger test and funnel plots.^[41]^

### 2.11 Date synthesis

We will use Review Manager 5.3 for all statistical analyses. The data will be pooled for the meta-analysis when the included studies are sufficiently homogeneous with respect to subjects, interventions, and outcomes. All similar studies will be pooled for a random-effects model to obtain the pooled intervention effect. The pooled intervention effect will be expressed in terms of the mean difference and 95% of CI if the outcome was reported as a continuous variable. If different scales were used to assess the outcome, the standard mean difference will be used. If the outcome was measured as a dichotomous variable, we will convert the OR into the standard mean difference as long as the underlying continuous measure followed an approximately normal distribution.

### 2.12 Subgroup analysis

To solve some potential problems, we will perform a subgroup analysis. We will compare the results of different types, dosages, and duration of antihistamine drugs and different dosage forms of CG.

### 2.13 Sensitivity analysis

To verify the robustness of the conclusions, a sensitivity analysis will be conducted to test the inclusion of low-quality studies in the meta-analysis and exclude the impact of studies with high or fuzzy bias risks.

### 2.14 Grading the quality of evidence

We will grade the recommendations, assessments, developments, and assessment methods of the working group to judge the quality of the evidence of the results. Risk of bias, consistency, directness, accuracy, publication bias, and additional points were the areas we assessed. The assessment results will be divided into 4 levels: high, moderate, low, or very low.

## 3 Discussion

CU is a high-incidence disease, easy to relapse. It has a serious impact on the quality of life and work of patients.^[4]^ However, simple antihistamines are only suitable for some patients.^[31]^ The symptoms of more than 70% of CU patients will continue for 2 to 5 years, 20% will last for more than 5 years,^[2,19,42,43]^ many patients are not satisfied with the current treatment because of the need for long-term medication.^[44]^ CG has been repeatedly tested clinically, and many studies at home and abroad in recent years have shown that it has a good effect on CU,^[45]^ especially the treatment of CG combined with antihistamine drugs. Not only improves the overall efficiency, also greatly reduces the adverse effects\response rate and recurrence rate.^[46–48]^ but there are also reports showing that long-term use of CG will produce adverse reactions.^[49]^ This article will provide a more comprehensive summary of the validity and safety of the existing evidence, hoping to provide convincing evidence for patients and clinicians in the decision-making process.

## Author contributions

**Conceptualization:** Wei Cao

**Data curation:** Xianjun Xiao, Leixiao Zhang, Ying Liu, Lu Wang, Yue Cao, Qianhua Zheng,

**Formal analysis:** Wei Cao, Siyuan Zhou.

**Investigation:** Lu Wang, Zihao Zou

**Methodology:** Wei Cao, Xianjun Xiao.

**Project administration:** Leixiao Zhang.

**Supervision:** Ying Li.

**Writing – original draft:** Wei Cao.

**Writing – review & editing:** Chunxiao Li, Ying Li.
